# A VP24-truncated isolate of white spot syndrome virus is inefficient in *per os* infection

**DOI:** 10.1186/s13567-017-0492-8

**Published:** 2017-12-11

**Authors:** Yali Han, Fang Li, Limei Xu, Feng Yang

**Affiliations:** 1 Key Laboratory of Marine Genetic Resources of State Oceanic Administration, Third Institute of Oceanography, Xiamen, China; 2 Laboratory for Marine Fisheries Science and Food Production Processes, Qingdao National Laboratory for Marine Science and Technology, Qingdao, China; 3 Fujian Key Laboratory of Marine Genetic Resources, Fujian Collaborative Innovation Center for Exploitation and Utilization of Marine Biological Resources, Xiamen, China; 4 State Key Laboratory Breeding Base of Marine Genetic Resources, Xiamen, China

## Abstract

White spot syndrome virus (WSSV) is a major pathogen of penaeid shrimp. Here we identified a new WSSV strain, WSSV-CN04, from naturally infected *Marsupenaeus japonicus*. Whole genomic sequencing results indicate that the WSSV-CN04 genome was 281 054 bp in length, and encoded 157 hypothetic proteins. The genome sequence of WSSV-CN04 was most closely related to the low-virulent strain WSSV-CN03, sharing 97.5% sequence identity. Notably, in WSSV-CN04, the major envelop protein VP24 was not only truncated but also absent in the virions. Since VP24 was previously reported to be essential for WSSV *per os* infection by mediating WSSV-chitin interaction, we further analyzed the peroral infection of WSSV-CN03 and -CN04 in *Litopenaeus vannamei*, and show that the infectivity of WSSV-CN04 was significantly lower than that of WSSV-CN03. When compared with WSSV-CN03-infected shrimp, fewer virions were detected in the digestive tract tissues of WSSV-CN04-infected shrimp at 4 hours post-infection (hpi), and the viral titers in the animals at 24 hpi were much lower. Moreover, a peptide corresponding to VP24 chitin-binding domain reduced the amount of WSSV-CN03 in the midgut to a level similar to that of WSSV-CN04 at 4 hpi. These findings indicate that the truncation of VP24 may attenuate the peroral infectivity of WSSV-CN04, and therefore verify the important role of VP24 in WSSV *per os* infection.

**Electronic supplementary material:**

The online version of this article (10.1186/s13567-017-0492-8) contains supplementary material, which is available to authorized users.

## Introduction

White spot syndrome virus (WSSV) is a major viral pathogen that affects shrimp aquaculture all over the world [1, 2]. WSSV is a rod-shaped enveloped virus typically 250 to 380 nm in length and 80 to 120 nm in diameter, containing a double-stranded DNA genome of ~300 kb [3]. The virus infects multiple crustacean species including penaeid shrimp, crayfish, crab and lobster [4–7]. In cultured penaeid shrimp, WSSV can cause up to 100% cumulative mortality within 3–10 days of infection [3]. Different WSSV isolates have been isolated from either penaeid shrimp or crayfish (Table 1), and the complete genome sequences have been determined for seven of them: WSSV-CN [8], WSSV-CN01, WSSV-CN02, WSSV-CN03 [9], WSSV-TH [10], WSSV-TW [11], WSSV-KR [12]. Since the genome of WSSV-EG3 (GenBank Accession Number KR083866) is identical to WSSV-CN, we do not consider it as a different strain.

**Table 1:** **Main characteristics of eight fully-sequenced WSSV genomes**

Strains	Size (bp)	G + C %	Year	Host	Accession no.	Identity (%)^a^
WSSV-CN04	281 054	41.0	2012	*Marsupenaeus japonicus*	KY827813	/
WSSV-CN	305 107	41.0	1996	*Marsupenaeus japonicus*	AF332093	92.0
WSSV-CN01	309 286	40.9	1994	*Marsupenaeus japonicus*	KT995472	90.4
WSSV-CN02	294 261	41.0	2010	*Procambarus clarkii*	KT995470	94.5
WSSV-CN03	284 148	41.0	2010	*Litopenaeus vannamei*	KT995471	97.5
WSSV-TW	307 287	41.0	1994	*Penaeus mondon*	AF440570	91.3
WSSV-TH	292 967	41.1	1996	*Penaeus mondon*	AF369029	94.6
WSSV-KR	295 884	41.0	2011	*Litopenaeus vannamei*	JX515788	94.2

^a^Identity in comparison with WSSV-CN04.

Because ingestion of WSSV-infected sick or dead shrimp is believed to be the major route of natural infection, the digestive tract of shrimp may be a primary site of infection [13–19]. The internal surfaces of shrimp esophagus, stomach, and hindgut are covered with chitinous linings [20, 21], while the food boluses in the midgut are surrounded by chitinous peritrophic membranes (PMs) [22–24]. To successfully infect the host, WSSV virions need to cross the PM in the midgut or the chitinous lining in the other parts of the digestive tract. In our recent work, the WSSV major envelope protein VP24 was identified as a chitin-binding protein. We showed that VP24 played an essential role in WSSV *per os* by mediating the interaction between WSSV and chitin structures in the digestive tract [25].

In the current study, we identified a novel WSSV isolate, WSSV-CN04, in naturally infected *Marsupenaeus japonicus*. Comparative genomic analysis shows that the genome of WSSV-CN04 is highly similar to that of WSSV-CN03. However, a major envelope protein, VP24, was truncated in the newly identified isolate. Therefore, the distribution of the truncated VP24 in WSSV-CN04 virions was analyzed, and the infectivity of WSSV-CN03 and -CN04 was compared.

## Materials and methods

### Animals

*Litopenaeus vannamei* (subadults) with an average body weight of 12 g were purchased from a local market in Xiamen, China. Juvenile *L. vannamei* with an average body weight of 12 mg were obtained from Xinrongteng Aquatic Technology Development Company. Shrimp were maintained in water tanks containing seawater with 10 ± 2‰ salinity at 25 °C with aeration. The animals were acclimatized for 7 days and fed with pelleted feed at a rate of 5% mean body weight per day. The animals were kept individually in plastic aquaria during the experiments.

Red swamp crayfish *Procambarus clarkii*, with an average body weight of 20 g, were purchased from a local market in Xiamen, and maintained in fresh water.

For subadult *L. vannamei* and *P. clarkii*, 20 μL hemolymph was withdrawn from each individual and subjected to fluorescence quantitative PCR (qPCR) analysis of WSSV genomic DNA (as described below). WSSV-free animals were used in the following experiments. For juvenile *L. vannamei*, 10% of the population were randomly selected for WSSV detection. If the 10% were WSSV-free, the rest of the population were used in the following experiments.

### WSSV stocks and purification of WSSV virions

The virus strain WSSV-CN04 was isolated from *M. japonicus* in Xiamen, China in 2012. The infected animals were stored at −80 °C until experimentation. To amplify the virus, 0.1 g muscle tissue of the infected animal was homogenized in 10 mL normal saline (0.9% NaCl) and then centrifuged at 5000 *g* for 5 min at 4 °C. The supernatant was filtrated through a 0.2 μm syringe filter, and then used to inoculate healthy *P. clarkii* at a dose of 100 μL/each. WSSV virions were purified from moribund *P. clarkii* and their concentrations were determined as described before [26, 27]. A portion of muscle tissue of each moribund *P. clarkii* was collected and the amount of virus in the tissue mass was measured by qPCR. Tissues with similar amounts of WSSV-CN03 and -CN04 were stored at −80 °C for feeding infection.

### Quantification of viral load by qPCR

The viral load in each sample was measured by qPCR using WSSV Fluorescent Quantitative PCR detection kit (Xiamen Lulong Biotech Co., Ltd., China). The amplification reactions were performed as follows: denaturing at 95 °C for 2 min, followed by 35 cycles at 94 °C for 10 s, and 60 °C for 30 s.

### Genome sequencing and analysis

WSSV genomic DNA was prepared from purified virions as described previously [28]. The viral genome was sequenced using 454 sequencing technology and assembled using the GS de novo assembler software (Version 2.8) by Shanghai Majorbio Bio-pharm Biotechnology Co., Ltd. The genome was annotated and analyzed using Geneious 10.0.5. The ORF of 60 aa or larger with minimum overlap were identified as potential protein coding genes. The genome structure was analyzed using the “align whole genomes” function of MAUVE [29]. The identities between the genomes were determined by the pairwise alignment of Geneious.

### SDS-PAGE, Coomassie blue staining and Western blot analysis

Purified WSSV virions were lysed in 1 × SDS-containing loading buffer, separated on 12% SDS-PAGE gel and stained with Coomassie brilliant blue. For Western blotting, proteins separated on SDS-PAGE were transferred to polyvinylidene difluoride (PVDF) membrane (Millipore). The blotting was performed using a Pierce G2 fast Blotter (Thermo). The membranes were blocked by incubating in Bløk™-CH reagent (Millipore) for 15 min at room temperature, followed by incubating with indicated primary antibodies for 15 min. The membranes were washed with TBST (50 mM Tris–Cl, 150 mM NaCl, 0.05% Tween 20, pH 7.5) and then incubated with alkaline phosphatase-conjugated goat anti-mouse IgG for 15 min. After three more washes with TBST, the signal was detected using the NBT/BCIP (nitroblue tetrazolium/5-bromo-4-chloro-3-indolylphosphate) substrate (Roche). For SDS-PAGE analysis, 1 × 10^9^ virions were loaded in each well. In Western blotting analysis, 1 × 10^8^ virions were loaded in each well to probe for VP26, VP24, or VP19, while 1 × 10^7^ virions were loaded in each well to probe for VP28. The mouse anti-VP28, and anti-VP26 monoclonal antibodies were produced by Shanghai Immune Biotech Ltd, China, and the mouse anti-VP24, and anti-VP19 polyclonal antibodies were generated by our lab.

### Transmission electron microscopy (TEM)

For TEM analysis, the suspension of purified virions was mounted onto formvar-coated, carbon-stabilized copper grids (200 mesh), and negatively stained with 1% sodium phosphotungstate (PTA, pH 7.0). The samples were observed using a transmission electron microscope (JEM-1230, JEOL).

### Challenge experiment for juvenile *L. vannamei*

Juvenile *L. vannamei* were maintained individually and starved for 24 h before challenge. The animals were randomly divided into three groups (20 individuals in each group). The infected muscle tissues were cut into strips of about 1 mm × 1 mm × 2 mm in size (~2 mg), and washed three times with clean sea water to remove free virions before use. The viral amounts in the tissues fed to the animals were determined by qPCR analysis of three randomly selected strips from the same individual. Then, each *L. vannamei* was fed with 2 mg WSSV-CN03 or -CN04 infected tissue (~1 × 10^6^ copies/mg tissue). The controls were fed with WSSV-free tissues. At 4 hpi and 24 hpi, 5 shrimp were randomly selected from each group for qPCR analysis. Each shrimp was weighed and homogenized in 10 volumes (wt/vol) of normal saline. Another 5 shrimp were collected from each group at 24 hpi for cryosectioning. The experiment was carried out in triplicate.

### Challenge experiment for subadult *L. vannamei*

*Litopenaeus vannamei* were starved for 24 h prior to inoculation. *S*hrimp were randomly divided into three groups (10 individuals per group), and inoculated with 2 × 10^9^ WSSV-CN04 virions in 30 μL normal saline, 2 × 10^9^ WSSV-CN03 virions in 30 μL normal saline, or 30 μL normal saline alone. Inoculum was delivered into the lumen of the esophagus using a 1-cm-long flexible silicone tube (diameter, 1.5 mm; wall thickness, 0.3 mm) as described before [25]. At 4 hpi, 5 shrimp were randomly selected from each group and the intact digestive tract of each shrimp was collected. The digestive tract tissues (including the esophagus, stomach, midgut, and hindgut tissues) were weighed and homogenized individually in 10 volumes (wt/vol) of normal saline for qPCR analysis. A section (~0.5 cm in length) of midgut was used for cryosectioning. The experiment was carried out in triplicate.

### Cryosectioning and immunofluorescence analysis

Samples were fixed overnight with 4% paraformaldehyde, dehydrated with 20% sucrose for 24 h, and 30% sucrose for 24 h, sequentially. For juvenile *L. vannamei*, the cephalothorax, and abdomen (about 0.5 cm in length) were collected from each animal. For subadult *L. vannamei*, a section of midgut about 0.5 cm in length was collected from each animal. The samples were placed in an optimal cutting temperature compound, transferred to liquid nitrogen, and stored at −80 °C prior to sectioning. The samples were cross-sectioned into 5-μm thick slices using a Leica CM1850 cryostat. The slices were dried in an oven overnight and sequentially fixed with cold acetone for 10 min, washed with PBS, probed with the anti-VP28 antibody, and incubated with the Alexa Fluor 488 donkey anti-mouse IgG secondary antibody (Life Technologies). The nucleus was stained with 4′,6-diamidino-2-phenylindole (DAPI). Tissue sections were observed with Confocal Laser Scanning Microscope Tcs SP5 (Leica), and fluorescence microscope DM6000B (Leica).

### In vivo protection assay with peptides

Peptide P-VP24_186–200_ (TNRHYLLSMRFSPGN) corresponding to the chitin-binding site of VP24 (aa 186–200) and the same-size control peptide P-VP24_148–162_ (GREFSANKFVLYFKP) from non-chitin binging region (aa 148–162 of VP24) were synthesized by Shanghai Science Peptide Biological Technology Co., Ltd. Subadult *L. vannamei* shrimp were randomly divided into five groups (5 individuals per group). For the negative-control group, each shrimp was infused with 30 μL normal saline. For the WSSV-CN03 infected group, the shrimp were inoculated orally with 2 × 10^9^ WSSV-CN03 virions plus 120 μg of P-VP24_186–200_ or P-VP24_148–162_ in 30 μL normal saline. For the WSSV-CN04 infected group, the shrimp were inoculated orally with 2 × 10^9^ WSSV-CN04 virions plus 120 μg P-VP24_186–200_ or P-VP24_148–162_ in 30 μL normal saline. The intact digestive tract was collected from each shrimp at 4 hpi. A section (~0.5 cm in length) of midgut was used for cryosectioning, and the rest of the digestive tract tissues were weighed and homogenized in 10 volumes (wt/vol) of normal saline for qPCR analysis. The experiment was carried out in triplicate.

### Statistics analysis

The qPCR data were subjected to independent-sample *t* test as indicated using PASW Statistics 18 software. *P* values of < 0.05 were considered statistically significant. *P < 0.05, **P < 0.01, ***P < 0.001.

## Results

### Genomic sequence of WSSV-CN04

The complete genomic sequence of WSSV-CN04 was determined, and deposited in the GenBank (Accession Number KY827813). The genome of WSSV-CN04 was assembled into a 281 054 bp circular molecule with 41.0% GC content and predicted to encode 157 hypothetical proteins (Additional file 1). The location, orientation, size, and function of each predicted protein-coding gene are summarized in Additional file 2. The main characteristics of eight fully-sequenced WSSV isolates are summarized in Table 1. The genomic sequence identities between WSSV-CN04 and the seven other WSSV isolates were determined by pairwise alignment; WSSV-CN04 shared the highest identity (97.5%) with WSSV-CN03.

### Comparative genomic analysis of WSSV-CN03 and -CN04

**Figure 1:**
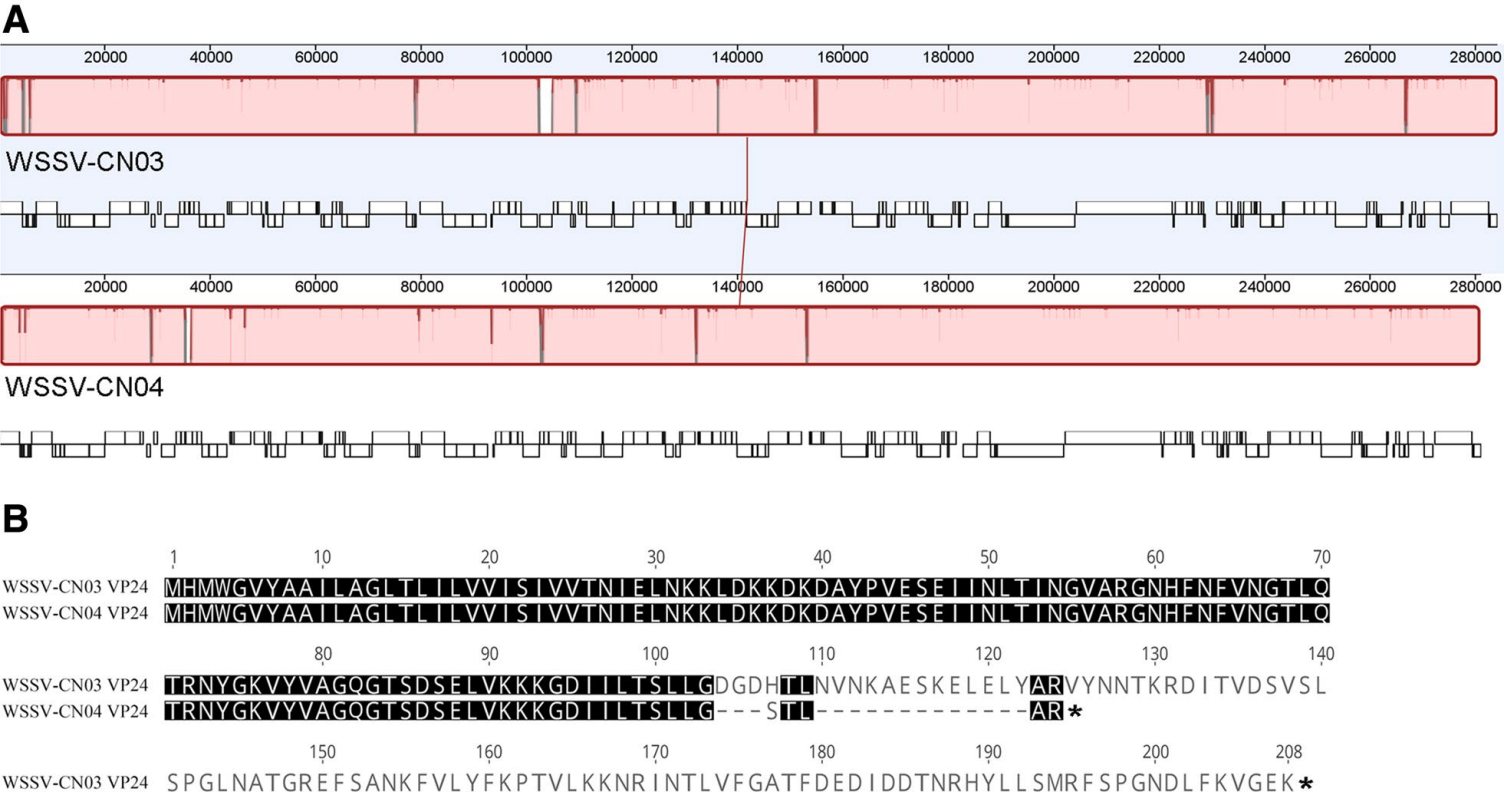
**Comparative genomic analysis of WSSV-CN03 and -CN04. A** The graphic result of the whole-genome alignment of WSSV-CN03 and -CN04. The genomic sequences of WSSV-CN3 and -CN04 were aligned with the “align whole genomes” function of MAUVE. Homologous blocks are shown as identically colored regions. ORF transcribed forward (above) and reverse (below) are indicated. **B** Schematic representation of VP24 variations. “*” indicates the stop codon.

Because the genome of WSSV-CN04 is most closely related to that of WSSV-CN03, the genomic sequences of WSSV-CN3, and CN04 were further aligned with the “align whole genomes” function of MAUVE (Figure 1A). The large majority of the genomic variations are present in the protein coding regions. The variations larger than 3 aa in the protein coding regions, including insertions, deletions, and substitutions were investigated and are summarized in Table 2. It is notable that the major envelope protein VP24 is truncated in WSSV-CN04 (Figure 1B). The intact VP24 protein contains 208 aa, but the C-terminus (aa 109–208) of the protein is missing in WSSV-CN04 due to a deletion mutation. Moreover, aa 104–108 is mutated in WSSV-CN04. This is the first time that a large-scale mutation is found in the major envelope proteins of WSSV.

**Table 2:** **Variations in the protein-coding genes of WSSV-CN03 and -CN04**

Genes	Function or structure	WSSV-CN03	WSSV-CN04	
		start–stop (aa)	Type of change	start–stop (aa)
wsv001	collagen-like	1–4371 (1456)	Del: 183 aa	1–3822 (1273)
wsv002	VP24, EP	5041–4415 (208)	Del: 100 aa	4216–3890 (108)
wsv006		6366–5461 (301)	Del: 34 aa	5439–4636 (267)
wsv073		Missing		35331–36599 (422)
wsv089		43202–43660 (152)	Del: 64 aa	43729–43995 (88)
wsv150		79052–78705 (115)	Ins: 32 aa	79701–79258 (147)
wsv195		Missing		102534–103304 (258)
wsv249	E3 ligase, IE	134655–136937 (760)	Del: 23 aa	132892–135105 (737)
wsv479		269533–269042 (163)	Ins: 126 aa	266439–265570 (289)
wsv486		104783–102447 (778)	Missing	/

aa: the number of predicted amino acids. IE: immediate early protein; EP: envelope protein; Del: deletion; Ins: insertion.

### VP24 is absent in the virions of WSSV-CN04

**Figure 2:**
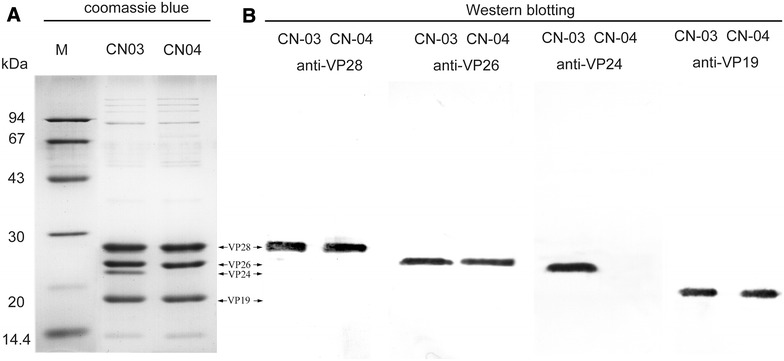
**SDS-PAGE and Western blot analysis of purified WSSV virions. A** Equal amounts of purified WSSV-CN03 and WSSVCN04 virions were lysed, separated on 12% SDS-PAGE gel, and stained with coomassie brilliant blue. **B** For Western blotting, proteins separated on SDS-PAGE gels were transferred to PVDF membranes. The membranes were probed with indicated primary antibodies, and then incubated with alkaline phosphatase-conjugated goat anti-mouse IgG. The signals were detected using the NBT/BCIP substrate.

VP28, VP26, VP24 and VP19 are four major envelope proteins that form a multi-protein complex together with some low abundant proteins. VP24 is supposed to be a core protein in this complex [30–32], which interacts with VP28, VP26, VP19, VP33, VP38, VP38A, VP51A, VP53A and wsv010 [30, 32–37]. To analyze whether C-terminal truncated VP24 affected the assembly of the protein in the virion, purified virions were lysed and analyzed by SDS-PAGE and Western blotting. In the SDS-PAGE analysis, the major envelope proteins VP28, VP26, VP24, and VP19 could be clearly detected in the lysate of WSSV-CN03, whereas the VP24 band was missing in the WSSV-CN04 lysate, and no extra band with lower molecular weight was observed (Figure 2A). Accordingly, in the Western blotting analysis, VP28, VP26 and VP19 were readily detected in both strains, while VP24 was absent in WSSV-CN04. No extra band with lower molecular weight was detected (Figure 2B). As the anti-VP24 polyclonal antibody was generated against full-length VP24 protein, the failure in VP24 detection should not be due to the loss of the epitope. Therefore, we deduce that the VP24 protein is not present in the virions of WSSV-CN04.

**Figure 3:**
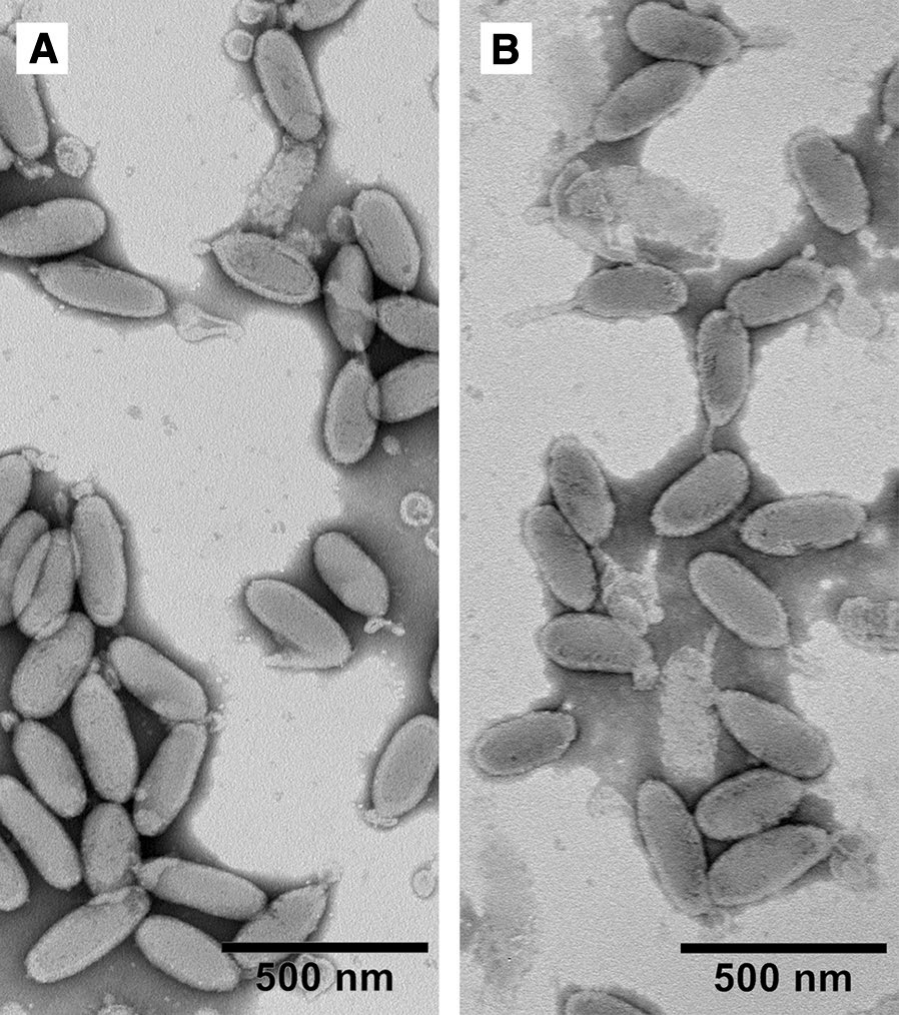
**Ultrastructural analysis of purified WSSV virions.** The suspension of purified virions was mounted onto formvar-coated, carbon-stabilized copper grids, and negatively stained with 1% sodium phosphotungstate. **A** WSSV-CN03; **B** WSSV-CN04.

Ultrastructural analysis shows that the virions of WSSV-CN03 and -CN04 were similar in size and shape. No obvious abnormal morphological features were observed in WSSV-CN04 virions (Figure 3). These data indicate that although VP24 can interact with quite a few high or low abundant structural proteins, it may not be essential for viral assembly. This is an unusual characteristic for a major structural protein of a virus.

### WSSV-CN04 is inefficient in *per os* infection

Oral ingestion is the major route for WSSV infection. In our previous work, VP24 has been demonstrated as a chitin binding protein that mediated the interaction between WSSV and the chitinous structure in the digestive tract, and was essential for WSSV *per os* infection [25]. Because VP24 is absent in WSSV-CN04 virions, we further investigated whether the infectivity of WSSV-CN04 was attenuated.

**Figure 4:**
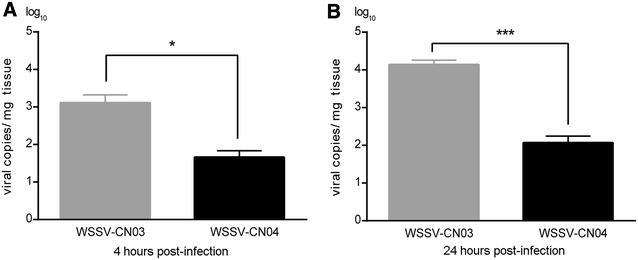
**qPCR analysis of the infection of WSSV-CN03 and -CN04 in juvenile** ***L. vannamei.*** *L. vannamei* were randomly divided into three groups. Each shrimp was fed with 2 mg WSSV-CN03-infected tissue or WSSV-CN04-infected tissue. The shrimp in the control group were fed with WSSV-free tissue. For each group, at 4 hpi and 24 hpi, 5 shrimp were randomly selected for q-PCR analysis. The columns represent the log base 10 of the mean WSSV copy numbers of 5 shrimp in each group, and the standard deviations were calculated. The data were subjected to independent-sample t test as indicated in figure. *P < 0.05; ***P < 0.001. The experiment was repeated three times and representative results from one experiment are shown.

**Figure 5:**
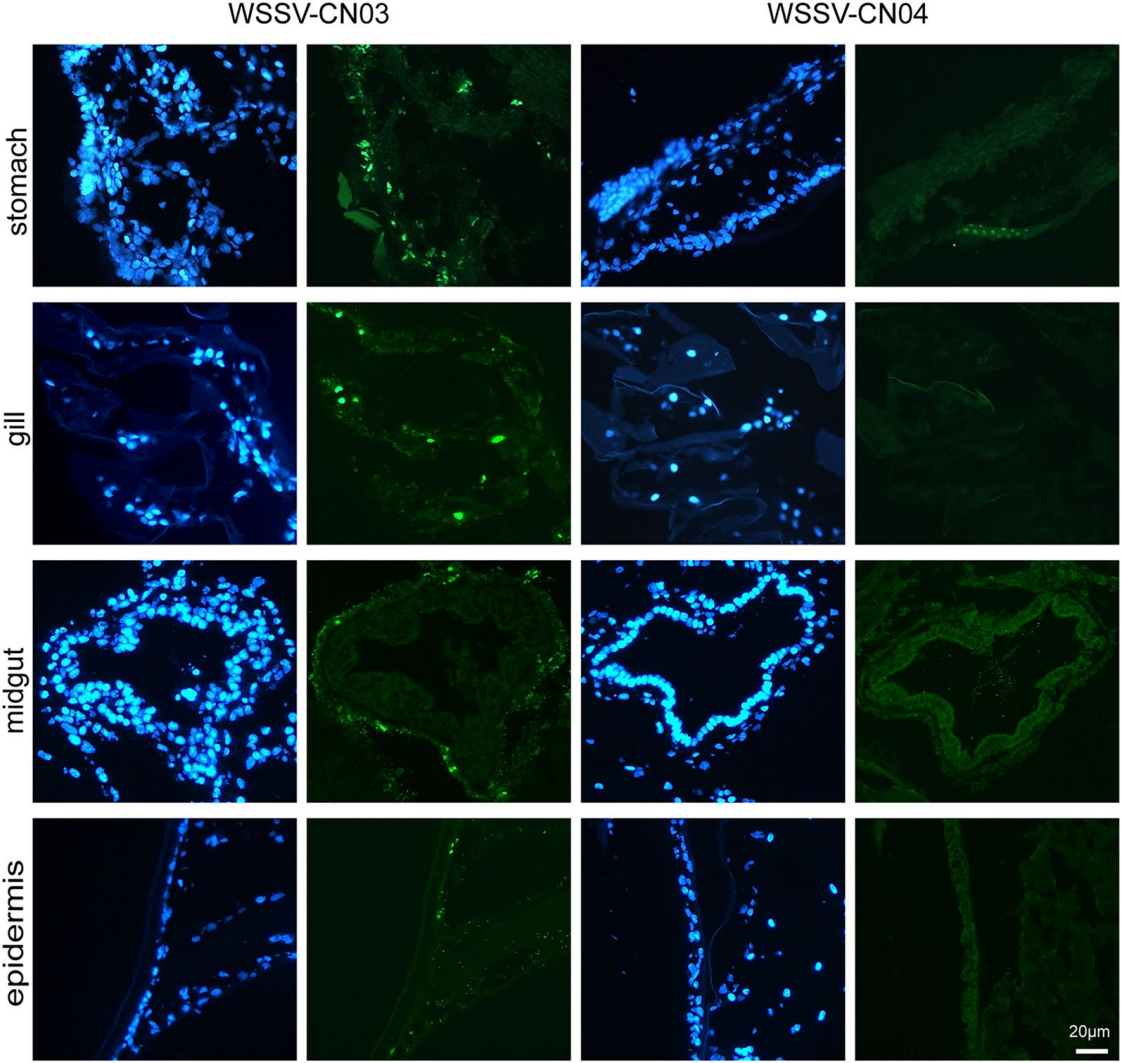
**Immunofluorescence analysis of WSSV-CN03 and -CN04 infection in juvenile** ***Litopenaeus vannamei*** *. L. vannamei* were randomly divided into three groups. Each shrimp was fed with 2 mg WSSV-CN03-infected tissue or WSSV-CN04-infected tissue. The shrimp in the control group were fed with WSSV-free tissue. At 24 hpi, 5 shrimp were randomly selected from each group for cryosectioning. The slices were probed with anti-VP28 antibody, and the nucleus was stained with DAPI. Bar 20 μm. The experiment was repeated three times and typical results of one experiment are shown.

The WSSV-CN03 or WSSV-CN04 infected tissues were cut into strips and fed to the juvenile *L. vannamei*. The animals in the control group were fed with WSSV-free tissue. The clearance time of ingested food from the entire digestive system has been estimated to be 4 h for *L*. *vannamei* [38]. Therefore, five animals were collected from each group at 4 hpi to evaluate the efficiency of WSSV binding to the digestive tract. qPCR analysis shows that the number of WSSV-CN04 virions remaining in the digestive tract at 4 hpi was 45 copies/mg tissue, which was significantly lower than that of WSSV-CN03 (1.3 × 10^3^ copies/mg tissue) (Figure 4A). Correspondingly, at 24 hpi, 10 animals were collected from each group to estimate viral replication. Five individuals were used for qPCR analysis and five were used for cryosectioning. The results show that the viral load in WSSV-CN04-infected animals (97 copies/mg tissue) was significantly lower than that in the animals infected with WSSV-CN03 (1.38 × 10^4^ copies/mg tissue) (Figure 4B). The difference was also clearly observed in the immunofluorescence analysis (Figure 5). In WSSV-CN03-infected shrimp, replication of the virus was detected in the nucleus of the cells in the stomach, gills, and cuticular epidermis. No WSSV-replication were observed in hepatopancreas cells (data not shown), muscle cells (data not shown), or midgut inner layer epidermis cells. In contrast, very few intracellular WSSV signals could be observed in WSSV-CN04 infected shrimp. These data imply that WSSV-CN04 *per os* infection is inefficient.

**Figure 6:**
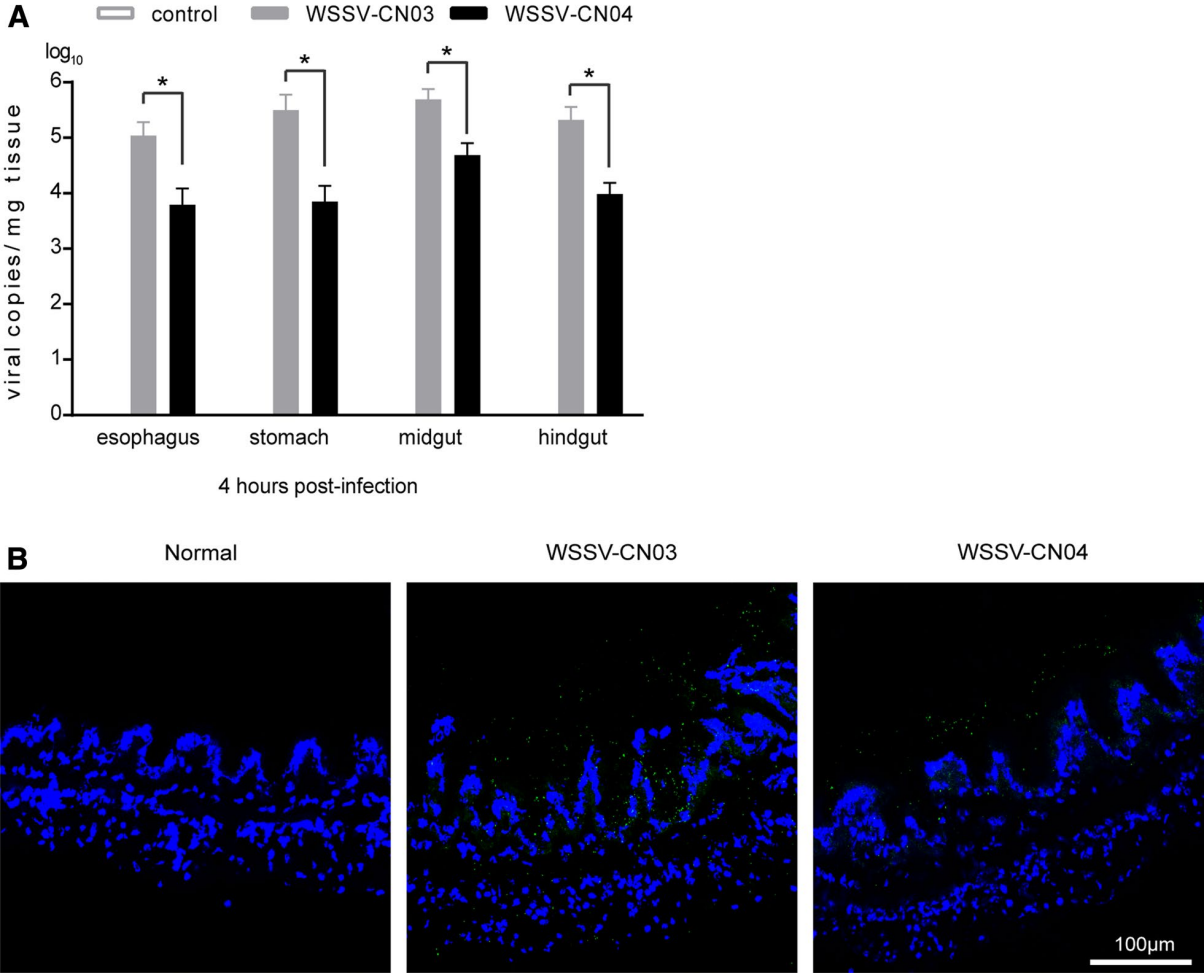
**Attachment of WSSV-CN03 and -CN04 to the inner surface of shrimp digestive tract.** Subadult *L. vannamei* were orally inoculated with WSSV-CN03, WSSV-CN04, or an equal volume of normal saline (negative control). The intact digestive tract of each shrimp was collected at 4 hpi, and the viral load in each tissue was analyzed via qPCR **A**. The columns represent the log base 10 of the mean WSSV copy numbers of 5 shrimp in each group, and the standard deviations were calculated. The data were subjected to independent-sample t test as indicated in figure using PASW Statistics 18 software. *P* values of ˂0.05 were considered statistically significant, and indicated with “*”. A small fragment of the midgut of each shrimp was cross-sectioned, and probed with an anti-VP28 antibody (green). The nucleus was stained with DAPI (blue). The inner surface of the midgut is facing upwards **B**. Bar 100 μm. The experiment was repeated three times and representative results from one experiment are shown.

Moreover, to investigate whether the infection deficiency was due to the weak interaction between WSSV-CN04 and the inner surface of the digestive tract, purified WSSV-CN03 or WSSV-CN04 virions were delivered to the oral cavity of subadult *L. vannamei*. Shrimp infused with normal saline were used as negative controls. The intact digestive tract was sampled from each animal at 4 hpi. A small segment of the midgut of each animal was used for cryosectioning, while the rest of the sample was used to determine viral load via qPCR. Compared with WSSV-CN04 infection group, the viral copy numbers in the esophagus, stomach, midgut and hindgut of WSSV-CN03-infected shrimp were 10–40 times higher (Figure 6A). Many viral particles (small green spots) could be observed in the midgut of WSSV-CN03-infected shrimp, either attaching to or penetrating through the inner layer (Figure 6B). Much fewer viral particles could be seen in WSSV-CN04-infected shrimp than in WSSV-CN03-infected shrimp. Therefore, the low infectivity of WSSV-CN04 through oral infection is very likely due to the poor attachment of virions to the inner surface of the digestive tract.

### Absence of VP24 attenuates WSSV-CN04 peroral infectivity

**Figure 7:**
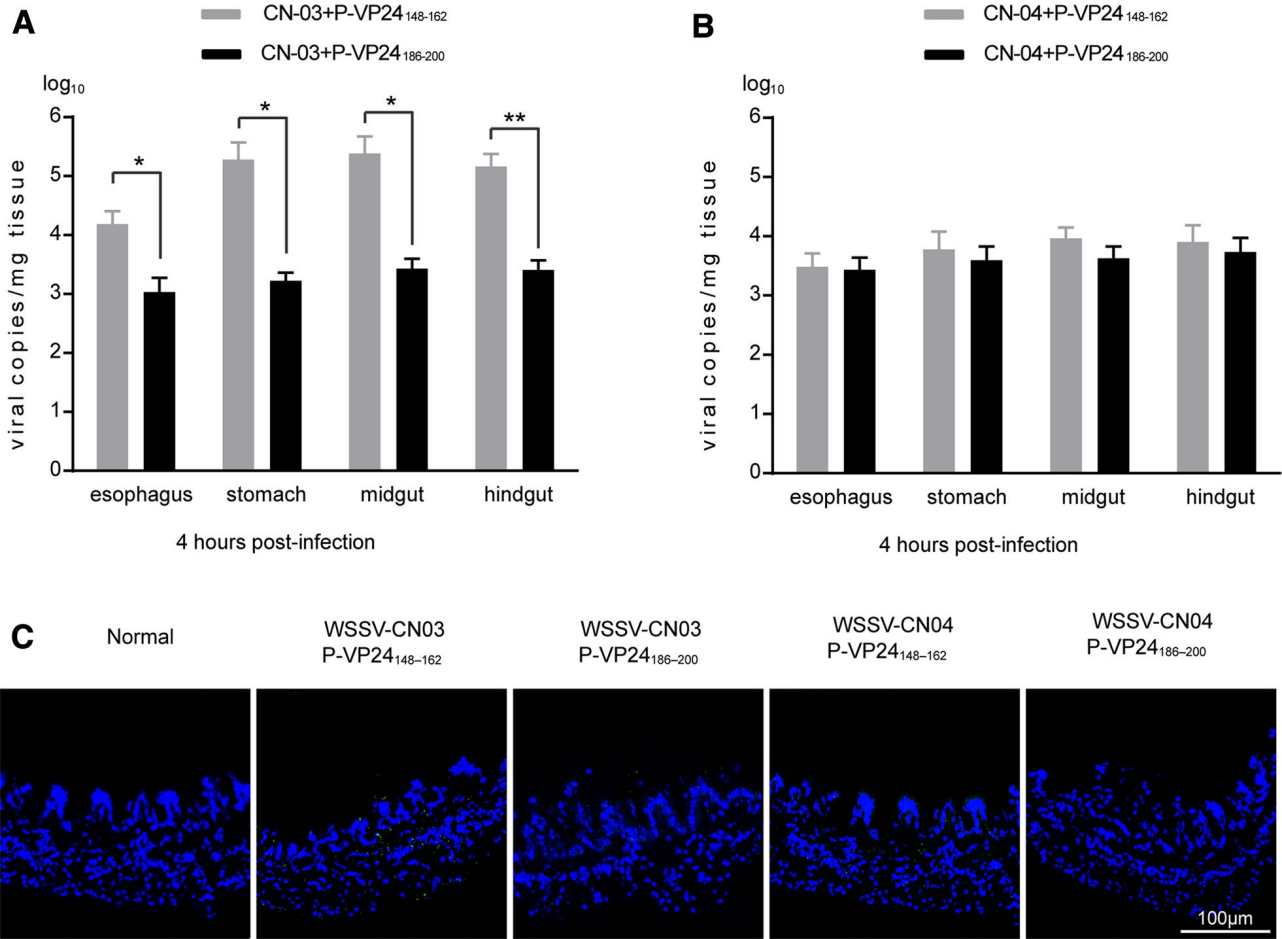
**Peptide inhibition assay.** Subadult *L. vannamei* shrimp were randomly divided into five groups (5 individuals per group). For the negative-control group, each shrimp was infused with 30 μL of normal saline. For the WSSV-CN03 infected groups, the shrimp were inoculated orally with 2 × 10^9^ WSSV-CN03 virions plus 120 μg of P-VP24_186–200_ or P-VP24_148–162_. For the WSSV-CN04 infected groups, the shrimp were inoculated orally with 2 × 10^9^ WSSV-CN04 virions plus 120 μg of P-VP24_186–200_ or P-VP24_148–162_. The tissues were collected from each group at 4 hpi for qPCR analysis (**A**, **B**). The data were subjected to independent-sample t test as indicated in figure. *P < 0.05; **P < 0.01. A section of midgut was used for cryosectioning (**C**) and probed with anti-VP28 antibody (green). The nucleus was stained with DAPI (blue). Bar 100 μm. The experiment was repeated three times and representative results from one experiment are shown.

To explore whether the attenuation of WSSV-CN04 peroral infectivity was related to the absence of VP24, a peptide inhibition assay was performed. Peptide corresponding to VP24 chitin-binding site (P-VP24_186–200_), which can block WSSV-chitin interaction [25], was delivered into the oral cavity of shrimp along with purified WSSV-CN03 or WSSV-CN04. Peptide P-VP24_148–162_ was used as a negative control. The viral loads in the esophagus, stomach, midgut and hindgut were measured at 4 hpi by qPCR. As shown in Figure 7 the attachment of WSSV-CN03 to the esophagus, stomach, midgut and hindgut was strongly inhibited by P-VP24_186–200_. However, the amounts of WSSV-CN04 in the digestive tract tissues were not significantly affected by the peptides. Notably, the viral loads of WSSV-CN03 in the digestive tract tissues after P-VP24_186–200_ treatment (Figure 7A) were similar to those of WSSV-CN04 (Figure 7B). These were coincident with the observation in immunofluorescence analysis. Therefore, the absence of VP24 in the virions may be a main reason for the deficiency of WSSV-CN04 *per os* infection.

## Discussion

In this study, we identified a new WSSV strain, WSSV-CN04, from naturally infected *M. japonicus*. The genome of this strain is highly similar to that of WSSV-CN03, sharing a sequence identity of 97.5% (Table 1). The most interesting variation is that the intact C-terminus of VP24 (aa 109–208) is truncated in WSSV-CN04 (Figure 1). This is the first time that a large-scale mutation was found in the major envelop proteins of WSSV, and as a result, this protein is lost from the virions (Figure 2). WSSV contains four major envelope proteins, VP28, VP26, VP24 and VP19, which are believed to form a multi-protein complex and recruit other low abundant proteins to form the WSSV “infectome”. VP24 was previously identified as the core of this WSSV envelope protein complex, and was supposed to be important for virus assembly [30–32]. However, in the current study, by analyzing the VP24-truncated strain WSSV-CN04, we demonstrate that the virions without obvious abnormality in morphology could be assembled in the absence of VP24 (Figure 3). Therefore, we speculate that VP24 may not be necessary for virus assembly or maintenance of the viral structure.

In our recent work, VP24 was identified as a chitin binding protein that mediates WSSV-chitin interaction, which makes it possible for the virus to attach and cross the chitinous barriers in the digestive tract [25]. Here by investigating the infection of WSSV-CN04 and -CN03 through feeding and oral inoculation, we showed that due to poor binding to the inner surface of the digestive tract (Figure 6), the peroral infectivity of WSSV-CN04 was significantly lower than that of WSSV-CN03 (Figures 4 and 5). Peptide inhibition assay with a peptide (P-VP24_186–200_) that specifically blocked VP24-chitin interaction proved that VP24-chitin interaction was essential for binding of WSSV-CN03 to the digestive tract. Moreover, the amount of WSSV-CN03 attached to the inner layer of the midgut in the P-VP24_186–200_-treated group decreased to a level similar to that of WSSV-CN04 (Figure 7). Therefore, the attenuation of WSSV-CN04 peroral infectivity is possibly due to the lack of VP24 in the virions. Instead of being a structure protein required for virus assembly, VP24 may mainly function in WSSV *per os* infection by mediating viral interaction with the inner layer of the host digestive tract and facilitating viral penetration through the chitinous barriers.

Notably, variation in replication speed may also be a reason for the change of viral infectivity. In some preliminary analysis, we found that when equal amounts of WSSV-CN03 and -CN04 were injected into crayfish, they proliferated at comparable speeds, and the animals survived for similar times (data not shown). This observation supports our hypothesis that the attenuation of WSSV-CN04 peroral infectivity is mainly due to the lack of VP24 in the virions, however, more experiments are required before making a definitive conclusion.

## Additional files

**Additional file 1.** **A schematic diagram showing the organization of the circular genome of WSSV-CN04.** Positions of the predicted protein coding genes and their transcription directions are indicated with arrows.

**Additional file 2.** **Predicted protein coding genes of WSSV-CN4. The genome of WSSV-CN04 was analyzed using Geneious 10.0.5.** The ORF of 60 aa or larger with minimum overlap were identified as potential protein coding genes. The location, orientation, size, and function of each predicted protein-coding gene are summarized.

## Abbreviations

WSSV: white spot syndrome virus
qPCR: quantitative polymerase chain reaction
aa: amino acid
PM: peritrophic membrane
PVDF: polyvinylidene difluoride
DAPI: 4′,6-diamidino-2-phenylindole

## References

[1] Shekar M, Pradeep B, Karunasagar I (2012). White spot syndrome virus: genotypes, epidemiology and evolutionary studies. Indian J Virol.

[2] Shekhar MS, Ponniah AG (2015). Recent insights into host–pathogen interaction in white spot syndrome virus infected penaeid shrimp. J Fish Dis.

[3] Leu J-H, Yang F, Zhang X, Xu X, Kou G-H, Lo C-F, Van Etten JL (2009). Whispovirus. Lesser known large dsDNA viruses.

[4] Kanchanaphum P, Wongteerasupaya C, Sitidilokratana N, Boonsaeng V, Panyim S, Tassanakajon A, Withyachumnarnkul B, Flegel TW (1998). Experimental transmission of white spot syndrome virus (WSSV) from crabs to shrimp *Penaeus monodon*. Dis Aquat Organ.

[5] Rajendran KV, Vijayan KK, Santiago TC, Krol RM (1999). Experimental host range and histopathology of white spot syndrome virus (WSSV) infection in shrimp, prawns, crabs and lobsters from India. J Fish Dis.

[6] Shi Z, Huang C, Zhang J, Chen D, Bonami JR (2000). White spot syndrome virus (WSSV) experimental infection of the freshwater crayfish, *Cherax quadricarinatus*. J Fish Dis.

[7] Hameed ASS, Yoganadhan K, Sathish S, Rasheed M, Murugan V, Jayaraman K (2001). White spot syndrome virus (WSSV) in two species of freshwater crabs (*Paratelphusa hydrodomous* and *P. pulvinata*). Aquaculture.

[8] Yang F, He J, Lin XH, Li Q, Pan D, Zhang XB, Xu X (2001). Complete genome sequence of the shrimp white spot bacilliform virus. J Virol.

[9] Li F, Gao M, Xu L, Yang F (2017). Comparative genomic analysis of three white spot syndrome virus isolates of different virulence. Virus Genes.

[10] van Hulten MCW, Witteveldt J, Peters S, Kloosterboer N, Tarchini R, Fiers M, Sandbrink H, Lankhorst RK, Vlak JM (2001). The white spot syndrome virus DNA genome sequence. Virology.

[11] Chen LL, Wang HC, Huang CJ, Peng SE, Chen YG, Lin SJ, Chen WY, Dai CF, Yu HT, Wang CH, Lo CF, Kou GH (2002). Transcriptional analysis of the DNA polymerase gene of shrimp white spot syndrome virus. Virology.

[12] Chai CY, Yoon J, Yong SL, Kim YB, Choi TJ (2013). Analysis of the complete nucleotide sequence of a white spot syndrome virus isolated from pacific white shrimp. J Microbiol.

[13] Soto MA, Shervette VR, Lotz JM (2001). Transmission of white spot syndrome virus (WSSV) to *Litopenaeus vannamei* from infected cephalothorax, abdomen, or whole shrimp cadaver. Dis Aquat Organ.

[14] Wang Q, White BL, Redman RM, Lightner DV (1999). *Per os* challenge of *Litopenaeus vannamei* postlarvae and *Farfantepenaeus* duorarum juveniles with six geographic isolates of white spot syndrome virus. Aquaculture.

[15] Wu JL, Namikoshi A, Nishizawa T, Mushiake K, Teruya K, Muroga K (2001). Effects of shrimp density on transmission of penaeid acute viremia in *Penaeus japonicus* by cannibalism and the waterborne route. Dis Aquat Organ.

[16] Chou HY, Huang CY, Lo CF, Kou GH (1998). Studies on transmission of white spot syndrome associated baculovirus (WSBV) in *Penaeus monodon* and *P. japonicus* via waterborne contact and oral ingestion. Aquaculture.

[17] Escobedo-Bonilla CM, Wille M, Sanz VA, Sorgeloos P, Pensaert MB, Nauwynck HJ (2005). In vivo titration of white spot syndrome virus (WSSV) in specific pathogen-free *Litopenaeus vannamei* by intramuscular and oral routes. Dis Aquat Organ.

[18] Di Leonardo VA, Bonnichon V, Roch P, Parrinello N, Bonami JR (2005). Comparative WSSV infection routes in the shrimp genera *Marsupenaeus* and Palaemon. J Fish Dis.

[19] Escobedo-Bonilla CM, Wille M, Sanz VA, Sorgeloos P, Pensaert MB, Nauwynck HJ (2007). Pathogenesis of a Thai strain of white spot syndrome virus (WSSV) in juvenile, specific pathogen-free *Litopenaeus vannamei*. Dis Aquat Organ.

[20] Felgenhauer BE, Harrison FWHA (1992). Internal anatomy of the Decapoda: an overview. Microscopic anatomy of invertebrates.

[21] Hackman RH, Wright JE, Retnakaran A (1987). Chitin and the fine structure of cuticles. Chitin and benzoylphenyl ureas.

[22] Eisemann CH, Binnington KC (1994). The peritrophic membrane: its formation, structure, chemical composition and permeability in relation to vaccination against ectoparasitic arthropods. Int J Parasitol.

[23] Tellam RL, Wijffels G, Willadsen P (1999). Peritrophic matrix proteins. Insect Biochem Mol Biol.

[24] Martin GG, Simcox R, Nguyen A, Chilingaryan A (2006). Peritrophic membrane of the penaeid shrimp *Sicyonia ingentis*: structure, formation, and permeability. Biol Bull.

[25] Li ZP, Li F, Han YL, Xu LM, Yang F (2016). VP24 Is a chitin-binding protein involved in white spot syndrome virus infection. J Virol.

[26] Xie XX, Li HY, Xu LM, Yang F (2005). A simple and efficient method for purification of intact white spot syndrome virus (WSSV) viral particles. Virus Res.

[27] Zhou Q, Qi YP, Yang F (2007). Application of spectrophotometry to evaluate the concentration of purified white spot syndrome virus. J Virol Methods.

[28] Yang F, Wang W, Chen RZ, Xu X (1997). A simple and efficient method for purification of prawn baculovirus DNA. J Virol Methods.

[29] Darling ACE, Mau B, Blattner FR, Perna NT (2004). Mauve: multiple alignment of conserved genomic sequence with rearrangements. Genome Res.

[30] Chang YS, Liu WJ, Lee CC, Chou TL, Lee YT, Wu TS, Huang JY, Huang WT, Lee TL, Kou GH, Wang AH, Lo CF (2010). A 3D model of the membrane protein complex formed by the white spot syndrome virus structural proteins. PLoS One.

[31] Li ZC, Xu LM, Li F, Zhou Q, Yang F (2011). Analysis of white spot syndrome virus envelope protein complexome by two-dimensional blue native/SDS PAGE combined with mass spectrometry. Arch Virol.

[32] Huang PY, Leu JH, Chen LL (2014). A newly identified protein complex that mediates white spot syndrome virus infection via chitin-binding protein. J Gen Virol.

[33] Chen J, Li Z, Hew CL (2007). Characterization of a novel envelope protein WSV010 of shrimp white spot syndrome virus and its interaction with a major viral structural protein VP24. Virology.

[34] Jie ZL, Xu LM, Yang F (2008). The C-terminal region of envelope protein VP38 from white spot syndrome virus is indispensable for interaction with VP24. Arch Virol.

[35] Zhou Q, Xu LM, Li H, Qi YP, Yang F (2009). Four major envelope proteins of white spot syndrome virus bind to form a complex. J Virol.

[36] Lin Y, Xu LM, Yang F (2010). Tetramerization of white spot syndrome virus envelope protein VP33 and its interaction with VP24. Arch Virol.

[37] Liu WJ, Shiung HJ, Lo CF, Leu JH, Lai YJ, Lee TL, Huang WT, Kou GH, Chang YS (2014). Characterization and interactome study of white spot syndrome virus envelope protein VP11. PLoS One.

[38] Marte CL (1980). The food and feeding habit of *Penaeus monodon* Fabricius collected from Makato River, Aklan, Philippines (Decapoda Natantia). Crustaceana.

