# Supplementary figures and images for: A VP24-truncated isolate of white spot syndrome virus is inefficient in per os infection
Source: Vet Res. 2017 Dec 11;48:87. doi: 10.1186/s13567-017-0492-8 (PMC5725807; doi:10.1186/s13567-017-0492-8)

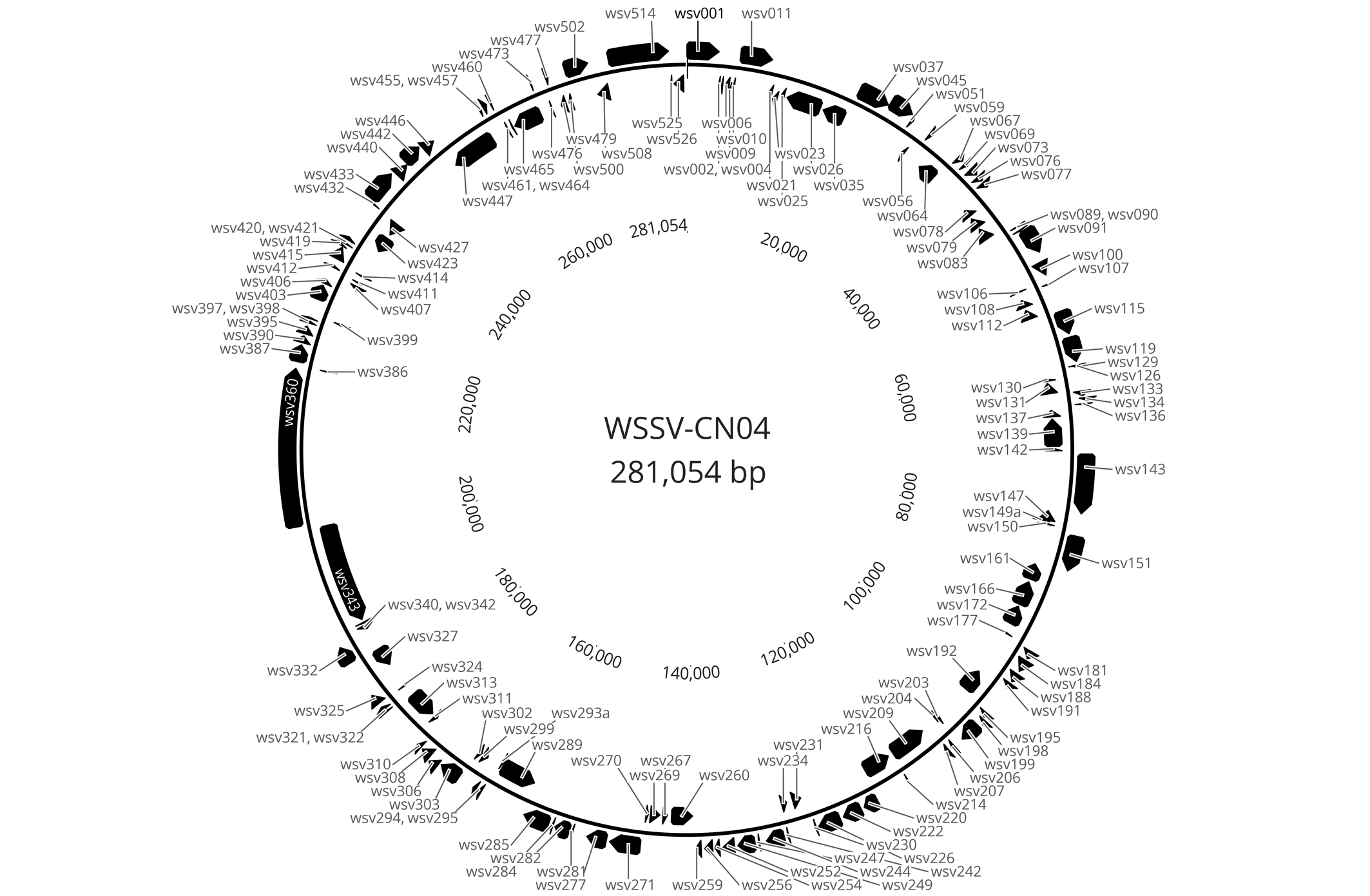

Supplement: Supplementary file 1 — Additional file 1. A schematic diagram showing the organization of the circular genome of WSSV-CN04. Positions of the predicted protein coding genes and their transcription directions are indicated with arrows. [file 13567_2017_492_MOESM1_ESM.tif]
