# Supplementary material for: A VP24-truncated isolate of white spot syndrome virus is inefficient in per os infection
Source: Vet Res. 2017 Dec 11;48:87. doi: 10.1186/s13567-017-0492-8 (PMC5725807; doi:10.1186/s13567-017-0492-8)
Supplement: Supplementary file 2 — Additional file 2. Predicted protein coding genes of WSSV-CN4. The genome of WSSV-CN04 was analyzed using Geneious 10.0.5. The ORF of 60 aa or larger with minimum overlap were identified as potential protein coding genes. The location, orientation, size, and function of each predicted protein-coding gene are summarized. [file 13567_2017_492_MOESM2_ESM.doc]

| Coding genes | Function or structure | start-stop (aa) |
| --- | --- | --- |
| wsv001 | collagen-like protein | 1-3822 (1273) |
| wsv002 | VP24, EP | 4216-3890 (108) |
| wsv004 |  | 4609-4295 (104) |
| wsv006 |  | 5439-4636 (267) |
| wsv009 | VP12, EP | 5686-5399 (95) |
| wsv010 |  | 6010-5717 (97) |
| wsv011 | VP150/VP53A, EP | 6065-9970 (1301) |
| wsv021 |  | 10659-10057 (200) |
| wsv023 |  | 11516-10656 (286) |
| wsv025 |  | 12262-11567 (231) |
| wsv026 |  | 16950-12343 (1535) |
| wsv035 | VP110, EP | 19998-17080 (972) |
| wsv037 | VP160B | 20015-23857 (1280) |
| wsv045 |  | 23799-26726 (975) |
| wsv051 | IE | 26707-27297 (196) |
| wsv056 | IE | 28574-27894 (226) |
| wsv059 |  | 29326-29952 (208) |
| wsv064 |  | 33244-30713 (843) |
| wsv067 | Thymidylate synthase | 33475-34344 (289) |
| wsv069 | IE1 | 34508-35182 (224) |
| wsv073 |  | 35331-36599 (422) |
| wsv076 |  | 36601-37431 (276) |
| wsv077 | VP36A, EP | 37457-38350 (297) |
| wsv078 | IE | 39628-38432 (398) |
| wsv079 | IE | 41300-39765 (511) |
| wsv083 | IE, protein kinase | 43098-41356 (580) |
| wsv089 |  | 43729-43995 (88) |
| wsv090 |  | 44123-44500 (125) |
| wsv091 | IE | 44457-47669 (1070) |
| wsv100 | IE | 48374-50248 (624) |
| wsv106 |  | 50713-50483 (76) |
| wsv107 |  | 51058-51369 (103) |
| wsv108 | IE | 52722-51502 (406) |
| wsv112 | dUTPase | 54234-52846 (462) |
| wsv115 | VP53B, EP | 54432-57338 (968) |
| wsv119 |  | 57478-60612 (1044) |
| wsv126 |  | 60736-61041 (101) |
| wsv129 |  | 60938-61201 (87) |
| wsv130 |  | 62063-61611 (150) |
| wsv131 |  | 63612-62155 (485) |
| wsv133 |  | 63689-64504 (271) |
| wsv134 |  | 64476-65144 (222) |
| wsv136 |  | 65151-65537 (128) |
| wsv137 |  | 66509-65496 (337) |
| wsv139 |  | 70144-66518 (1208) |
| wsv142 |  | 70603-70190 (137) |
| wsv143 |  | 70750-77697 (2315) |
| wsv147 |  | 79144-77765 (459) |
| wsv149a |  | 79352-78939 (137) |
| wsv150 |  | 79701-79258 (147) |
| wsv151 |  | 80047-84357 (1436) |
| wsv161 |  | 86689-84410 (759) |
| wsv166 |  | 89962-86744 (1072) |
| wsv172 | RR1 | 92589-90043 (848) |
| wsv177 |  | 93805-93626 (59) |
| wsv181 |  | 94087-95205 (372) |
| wsv184 |  | 95207-96832 (541) |
| wsv188 | RR2 | 97011-98252 (413) |
| wsv191 | nuclease | 98317-99252 (311) |
| wsv192 |  | 102348-99289 (1019) |
| wsv195 |  | 102534-103304 (258) |
| wsv198 | VP32, EP | 103304-104140 (278) |
| wsv199 | E3 ligase | 104220-106793 (857) |
| wsv203 |  | 107154-106615 (179) |
| wsv204 |  | 107447-107058 (129) |
| wsv206 |  | 107762-108376 (204) |
| wsv207 |  | 108473-109297 (274) |
| wsv209 | VP187, EP | 114159-109342 (1605) |
| wsv214 | VP15, DNA-binding protein | 114316-114501 (61) |
| wsv216 | VP124, EP | 118196-114609 (1195) |
| wsv220 | VP76/VP73, CP | 118266-120290 (674) |
| wsv222 | E3 ligase | 120309-122843 (844) |
| wsv226 |  | 122967-125783 (938 ) |
| wsv230 | ICP11/P9 | 125987-126235 (82) |
| wsv231 |  | 127782-126391 (463) |
| wsv234 |  | 129149-128265 (294) |
| wsv242 | VP41B, EP | 129014-129457 (147) |
| wsv244 |  | 129530-131905 (791) |
| wsv247 |  | 132493-132732 (79) |
| wsv249 | E3 ligase, IE | 132892-135105 (737) |
| wsv252 |  | 135276-136781 (501) |
| wsv254 | VP33/V36B/VP37, EP | 136861-137706 (281) |
| wsv256 | VP52B/P51B, EP | 137710-138864 (384) |
| wsv259 | VP38/VP38A, EP | 138925-139854 (309) |
| wsv260 |  | 142682-139914 (922) |
| wsv267 |  | 143777-142932 (281) |
| wsv269 | VP53C | 145310-143841 (489) |
| wsv270 |  | 145840-145328 (170) |
| wsv271 | VP136/ VP136A, CP | 145853-149506 (1217) |
| wsv277 |  | 149719-152094 (791) |
| wsv281 |  | 153633-153842 (69) |
| wsv282 |  | 154002-155906 (634) |
| wsv284 | VP13A | 155913-156215 (100) |
| wsv285 |  | 156368-159670 (1100) |
| wsv289 | VP190/VP160A, CP | 164464-159767 (1565) |
| wsv293a | VP14, EP | 164711-164418 (97) |
| wsv294 |  | 164764-165384 (206) |
| wsv295 |  | 165483-166112 (209) |
| wsv299 |  | 167090-166161 (309) |
| wsv302 |  | 167726-167157 (189) |
| wsv303 | TBP | 167829-170504 (891) |
| wsv306 | VP39A, EP | 170491-171750 (419) |
| wsv308 | VP51/VP51C/VP466, CP | 171775-173175 (466) |
| wsv310 |  | 173181-173,999 (272) |
| wsv311 | VP26, EP | 174687-174073 (204) |
| wsv313 |  | 178471-174926 (1181) |
| wsv321 | VP16/VP13B, EP | 178786-179142 (118) |
| wsv322 |  | 179156-179836 (226) |
| wsv324 |  | 180088-179840 (82) |
| wsv325 | VP56/VP60A, EP | 180087-181484 (465) |
| wsv327 | VP90, EP | 185397-182827 (856) |
| wsv332 | VP75 | 185530-187890 (786) |
| wsv340 | VP31, EP | 188786-188001 (261) |
| wsv342 |  | 189191-188,889 (100) |
| wsv343 |  | 201848-189294 (4184) |
| wsv360 | VP664, CP | 202122-220337 (6071) |
| wsv386 | VP12B | 220684-220478 (68) |
| wsv387 |  | 220863-223052 (729) |
| wsv390 | anti-apoptosis protein | 223105-224073 (322) |
| wsv395 | TK-TMK | 224094-225290 (398) |
| wsv397 |  | 225310-225825 (171) |
| wsv398 |  | 225874-226257 (127) |
| wsv399 |  | 226620-226270 (116) |
| wsv403 | IE | 228198-230123 (641) |
| wsv406 |  | 230178-230957 (259) |
| wsv407 |  | 231658-230975 (227) |
| wsv411 |  | 232135-231941 (64) |
| wsv412 |  | 232232-232711 (159) |
| wsv414 | VP19, EP | 233156-232791 (121) |
| wsv415 | VP60/VP60B, CP | 233293-234927 (544) |
| wsv419 |  | 234735-235316 (193) |
| wsv420 |  | 235303-235596 (97) |
| wsv421 | VP28, EP | 235760-236374 (204) |
| wsv423 | protein kinase | 238661-236469 (730) |
| wsv427 | latency-related | 240748-238877 (623) |
| wsv432 |  | 240669-240977 (102) |
| wsv433 |  | 240944-244729 (1261) |
| wsv440 |  | 244815-246638 (607) |
| wsv442 | VP95, EP&CP | 246593-248992 (799) |
| wsv446 |  | 249,067-250650 (527) |
| wsv447 |  | 256487-250683 (1934) |
| wsv455 |  | 256591-257112 (173) |
| wsv457 |  | 257118-257915 (265) |
| wsv460 |  | 257986-258402 (138) |
| wsv461 |  | 258732-258439 (97) |
| wsv464 |  | 259276-258923 (117) |
| wsv465 | VP136B, IE | 263112-259384 (1242) |
| wsv473 |  | 263140-263412 (90) |
| wsv476 |  | 264613-264398 (71) |
| wsv477 |  | 264890-265516 (208) |
| wsv479 |  | 266439-265570 (289) |
| wsv500 |  | 267260-266631 (209) |
| wsv502 |  | 267160-270189 (1009) |
| wsv508 |  | 271852-270236 (538) |
| wsv514 | DNA pol | 272276-279331 (2351) |
| wsv525 |  | 279587-279372 (71) |
| wsv526 |  | 280986-279640 (448) |

aa: The number of predicted amino acids ; IE: immediate early protein; EP: envelope protein; CP: capsid protein; RR1: Ribonucleotide reductase large subunit; RR2: Ribonucleotide reductase small subunit; TBP: TATA-box binding protein; TK-TMK: Chimeric Thymidine kinase-Thymidylate kinase.
